# Conduct disorders and psychopathy in children and adolescents: aetiology, clinical presentation and treatment strategies of callous-unemotional traits

**DOI:** 10.1186/s13052-017-0404-6

**Published:** 2017-09-20

**Authors:** Simone Pisano, Pietro Muratori, Chiara Gorga, Valentina Levantini, Raffaella Iuliano, Gennaro Catone, Giangennaro Coppola, Annarita Milone, Gabriele Masi

**Affiliations:** 1 Department of Medicine and Surgery, Clinic of Child and Adolescent Neuropsychiatry, University of Salerno, Baronissi, SA Italy; 2 IRCCS Stella Maris, Scientific Institute of Child Neurology and Psychiatry, Calambrone, Pisa, Italy; 3 Department of Mental and Physical Health and Preventive Medicine, Child and Adolescent Psychiatry Division, University of Studies of Campania “Luigi Vanvitelli”, Naples, Italy; 4 Department of Pediatrics, Hospital “F. Veneziale”, Isernia, Italy

**Keywords:** Conduct disorder, Callous unemotional traits, Psychopathy, Children, Adolescents

## Abstract

Conduct Disorder (CD) is a psychiatric diagnosis characterized by a repetitive and persistent pattern of behaviour in which the basic rights of others and major age-appropriate social norms or rules are violated. Callous Unemotional (CU) traits are a meaningful specifier in subtyping CD for more severe antisocial and aggressive behaviours in adult psychopathology; they represent the affective dimension of adult psychopathy, but they can be also detected in childhood and adolescence. The CU traits include lack of empathy, sense of guilt and shallow emotion, and their characterization in youth can improve our diagnostic, prognostic and therapeutic abilities. A strong genetic liability, in interaction with parenting and relevant environmental factors, can lead to elevated levels of CU traits in children. We pointed out that CU traits can be detected in early childhood, may remain stable along the adolescence, but a decrease following intensive and specialized treatment is possible. We here provide a narrative review of the available evidences on CU traits in three main domains: aetiology (encompassing genetic liability and environmental risk factors), presentation (early signs and longitudinal trajectories) and treatments.

## Background

Conduct Disorder (CD) is a psychiatric diagnosis characterized by a repetitive and persistent pattern of behaviour in which the basic rights of others and major age-appropriate social norms or rules are violated. CD is among the most frequent clinical conditions in child and adolescent mental health [1], with a host of social, emotional, and behavioural problems with high costs for the community. Aggressive behaviours towards other persons or animals or properties, as well as deceitfulness, theft or other severe violations of rules are the core features of this disorder [2]. This category, although necessary for a diagnosis, cannot capture the complexity of the clinical manifestations of CD, which are highly heterogeneous in terms of clinical presentation (high or low level of socialized behaviours, early or late onset, with impulsive or proactive aggression, high or low rate of comorbid affective disorders), outcome (remission or chronicity) and response to treatments (good or poor response to psychoterapy or pharmachotherapy) [3]. The fifth edition of Diagnostic and Statistical Manual of Mental Health [2] (DSM-5) has defined a new specifier for the CD diagnosis named “with limited prosocial emotions” (LPE); this update should help clinicians and researchers in reducing this heterogeneity, to improve their diagnostic and prognostic abilities [4, 5].

Psychopathic traits, previously considered as a meaningful (negative) specifier for severe antisocial and aggressive behaviours in adult psychopathology, have been re-discovered as a relevant factor in subtyping CD in youth [6]. The current concept of psychopathy comes from Hervey Cleckley’s seminal paper, describing a pattern of personality characterized by low levels of empathy and sense of guilt, arrogance, superficial charm, irresponsible and resulting antisocial behaviours [7]. Subsequent research, based on latent class analysis, better disentangled this issue in three conceptually separated, but inter-correlated domains: an interpersonal domain, consisting of grandiose-manipulative traits; an affective domain, consisting of callous unemotional (CU) traits; and a behavioural domain, consisting of daring-impulsive traits [8]. The grandiose-manipulative domain (also named narcissism) is characterized by verbal and manipulative abilities, superficial charm, egocentricity and glibness. The callous unemotional traits consist of lack of empathy and remorse, with short-lived emotions. The daring-impulsive domain (also named impulsivity or psychopathy-related impulsivity) traits include irresponsibility, proneness to boredom, novelty seeking and antisocial behaviour. Previous research pointed out that these domains are not exclusive of adult psychopathology, but they can also be found in children and adolescents [9], leading to the concept of childhood psychopathy, firstly conceptualized by Forth and colleagues [10], and further supported by Frick [11] and Lynam [12]. Subsequent studies [6, 13] confirmed that the multidimensional structure of adult psychopathy is detectable also in the adolescent populations. Furthermore, these studies on children/adolescents strongly suggested the association between psychopathic personality traits and conduct problems *(CP)*, namely aggression and law violation [14–16]. As said before, the, DSM-5 [2] has consolidated this connection, considering the callous unemotional (CU) traits as the LPE specifier for the CD. This specifier, defining a specific subtype of patients with CD, includes symptoms such as lack of remorse or guilt, callous lack of empathy, lack of concern about performance, and shallow or deficient affect. About 12 to 46% of youth with CD show significant CU traits [17–20]. The presence of CU traits in children and adolescents with CD defines a subclass of children characterized by a poorer adolescence outcome both in clinical and control samples [21], with increased risk for developing psychopathy in adulthood, with severe and persistent antisocial behaviour [3].

However, these studies raise the issue of translating the measures of CU traits into DSM-5 criteria of CD with LPE. According to DSM-5, CU traits should be present “persistently over at least 12 months and in more than one relationship or setting” [2]. Therefore, it is important to verify whether these features reflect the child’s typical pattern of interpersonal and emotional functioning, and not sporadic occurrences in specific situations*.*

Disentangling CD patients through an evaluation of the presence of CU traits is a crucial component of the assessment. However, clinical assessment of CU traits may present specific caveats. Firstly, the stability of CU traits is not well established, as not all the children presenting these characteristics at the first evaluation will continue to show them across childhood, and until early adolescence [22–24]. Many factors may be implicated in the development and maintenance of the CU traits, although it is hard to separately test the specific contribution of each element, i.e., genetic vs. environmental, to the onset and progression of these traits. Children grow up with their biological parents, thus they receive the genetic heritage, but at the same time, they are exposed to parents’ personality traits as well as to parenting strategies.

**Fig. 1:**
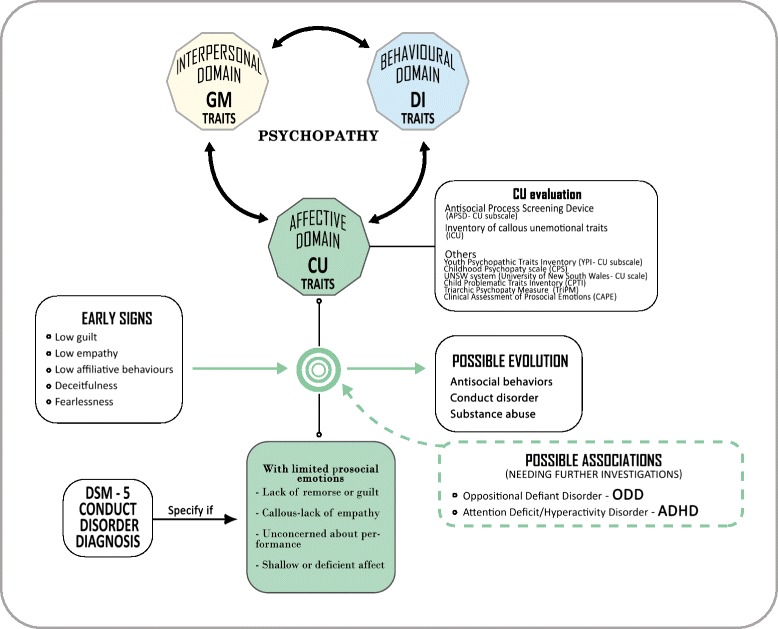
Graphical representation of the concept of developmental psychopathy, from a personality-based psychopathology (upper side) as well as a DSM-5 oriented (lower side) perspectives. GM: grandiose-manipulative, DI: daring-impulsive, CU: callous-unemotional

In the following sections, we narratively summarize latest findings about the concept of psychopathy in childhood, with a specific focus on their translation into clinical work of health professionals facing with youth with severe behavioural problems. The review will specifically focus on CU traits, given their presence as specifier in DSM-5 criteria of CD. Neuro-cognitive markers, genetic and environmental influences on CU onset will be explored, as well as their implications on developmental trajectories and treatment strategies. We will not cover neurobiological markers of CD and CU traits or ways of assessment, and refer readers to other reviews [9, 25, 26]. See Fig. 1 for a graphical representation of the concept of psychopathy in children, from a personality-based as well as from a DSM-5 oriented perspectives. In the figure have also been resumed the main instruments (self- or parent- report questionnaires) for the evaluation of CU traits in childhood/adolescence; for an extensive review see the work of Masi et al. [26].

## Neurocognitive issues

Previous studies showed that altered neuro-cognitive functions (e.g. intelligence, attentional network, processing of external stimuli) may underlie dysfunctional personality disorder features [27]. Similarly, many studies have documented neuro-cognitive correlates of psychopathy in children and adolescent.

In regard to intelligence, it is generally accepted that grandiose manipulative (narcissism) domain is linked to better performances in several cognitive areas, whereas CU traits were negatively associated with verbal intelligence, creativity, practicality and analytic thinking; finally, the daring-impulsive dimension was positively associated with measures of creativity, practicality, and analytic thinking, but not with verbal abilities [28, 29].

In their extensive review Frick et al. [9] pointed out that youth with CU traits show abnormalities in the processing of punishment cues and in the affective empathy (whereas data on cognitive empathy are more controversial); also, there are clear meta analytic evidences that antisocial personality is characterized by impairment in the facial recognition of fear and sadness [30], but also to other emotions and other way of expression (e.g. vocal) [31]. Furthermore, it is evident that high CU traits are associated with a less aversive way to interpret neutral and negative stimuli [32]. It has been suggested that the levels of CU traits are also associated with a generalized impairment in the natural allocation of attention to emotionally salient stimuli that results in cascading errors in recognizing other people’s emotions [33]. All these findings indicate an altered way of processing emotions and external stimuli and may explain why negative reinforcement could fail in the treatment process [32].

## Factors contributing to the onset and development of CU traits

In 2014 Frick et al. [34], reviewed nine publications about the heritability of CU traits and found that genetically accounted variations of CU traits ranged from 42% to 68% [35–39], and that a great part of the stability in CU traits during development is genetically driven [36, 40, 41]. These studies also indicate that, in front of a large shared genetic effect between the CD and the CU traits, the coexistence of specific genetic influences for both the constructs suggests the existence of a partially specific genetic aetiology [35, 37, 38, 42, 43].

Other factors that may contribute to the onset and the development of CU traits are represented by parents’ temperament and personality traits and by the parent-child interaction.

Two recent studies involving 561 adopted children and their biological mothers and adoptive families explored the first critical issue [44, 45]. In the first study [44], temperament (fearlessness) of the biological mother predicted CU behaviour of the adopted child at 27 months, via earlier fearlessness measured at 18 months; similarly, low affiliative behaviour of biologic mothers directly predicted child CU behaviours, although without any correlation with child affiliative behaviours tested at 18 months. Furthermore, adoptive mothers with highly positive parenting buffered the risk for developing CU traits via fearlessness. In the second study [45], a specific heritable pathway to CU behaviour was suggested: antisocial behaviour of the biological mother predicted CU behaviours of the child in late toddler period. Furthermore, more positively reinforcing mothers buffered inherited risk for early CU behaviours. These relevant studies strongly suggest that positive parenting has an effect on buffering genetic predisposition to develop CU traits.

The relation between parenting and CU traits development is bidirectional [46]. If the association between some features of dysfunctional parenting and the presence of CU traits in offspring has been supported by previous research, on the other hand children showing high CU traits can induce negative modifications in parenting behaviour. Although few studies specifically explored these mutual influences, preliminary evidences suggest that CU traits may be more predictive of changes in parenting over time than parenting on changes in CU traits [47, 48]. Some studies indicated that the presence of CU traits in children with CD is associated with low warmth in parenting [49, 50], others found harsh, inconsistent, and coercive discipline to be highly associated with CP in youth with normative levels of CU traits [50–55]. Viding, Fontaine, Oliver, & Plomin [56], in their study on monozygotic twins, found that negative parental discipline operates as a non-shared environmental risk factor for developing CP during the transition to early adolescence, but not for the development of CU traits.

However, studies investigating the association between quality of parenting and prospective change in CU traits in preschool samples have found that multiple domains of parenting (positive parenting, parental involvement, and poor monitoring/supervision) uniquely predicted changes in CU traits [47], and that high CU traits at ages 3–4 were predicted by parent harshness [57]. Other studies have investigated the direct prediction of CU traits by parenting in middle and late childhood. Loney, Huntenburg, Counts-Allan, and Schmeelk [58] assessed the intergenerational continuity of psychopathic traits, and found that the association was mediated by parenting. Parental-reported corporal punishment and child-reported parental warmth/involvement predicted CU traits, as children with low levels of anxiety who reported low parental warmth showed increased CU features 1 year later [59]. Other studies have related an increase in psychopathic characteristics to parental psychological aggression and inconsistency [60], or to poor supervision, physical punishment, and poor parent-child communication [61], or to maternal reports of harsh parenting at age 4 [62]. On the contrary, Vitacco, Neumann, Ramos, and Roberts [63] found that poor parental monitoring and inconsistent discipline were both related to impulsivity and narcissism, but not to CU traits.

In a recent publication exploring developmental trajectories of CU traits, youth with persisting high CU traits had experienced more maternal harshness, low parental knowledge, and monitoring compared to the moderate or low CU youth groups [64]. More specifically, harshness increased significantly across groups from low to high CU traits, and youth with low CU traits reported higher parental warmth, but there were no differences between the warmth reported by high compared to moderated groups.

In a study about effects of positive and negative parenting practices on the levels of child CU in 126 Italian children treated by a multi-modal approach including parent-training, Muratori et al. [65] found that increased levels of positive parenting predicted a decrease in the levels of CU traits in children. In addition, low levels of CU traits at the intermediate evaluation promoted an increase of positive parenting 1 year later. In contrast to previous studies [56], they found that negative parenting practices are unrelated to the level of CU traits when positive parenting is taken into account. Finally, Waller, Shaw and Hyde [66] observed that the presence of fearlessness at 24 months predicted CU behaviours at 42 months, but only when parents exhibited low levels of positive parenting. Furthermore, they showed that this association had a lasting effect on the presence of CU behaviour at 11–12 years. In summary, all these data suggest that the trajectory of children at high risk for CU behaviours could be modified by high levels of positive parenting [67].

In addition to parenting, other environmental and social factors can influence the development of CU traits and, in turn, parenting itself can be influenced by social context (as it as been theorized in 1984 by Belsky [68]). Recently, Waller et al. [67] applied Belsky’s model in relation to the development of CU behaviour. They found that parental warmth at age 2 uniquely predicted parent-reported CU behaviour at ages 10–12, controlling for both concurrent AB and early contextual risk factors across domains [69]. In addition, there was a direct association between neighborhood impoverishment and higher CU behaviour at ages 10–12 and age 20. Moreover, contextual risk and maternal characteristics were indirectly linked to later child CU behaviour by shaping less warm parenting style. These data strengthen the concept that a negative parenting style may enhance the developing of CU behaviour and/or AB, particularly among families with low socio-economic status [46, 67].

Tuvblad and colleagues’ study [70] on genetic and environmental determinants of the psychopathic personality in a community sample of 5-year-old twins indicated that both genetic and shared environmental influences are of importance for psychopathy personality traits in childhood. Regarding the CU dimension, they found a moderate (25%) genetic influence and a higher (48%) shared environmental influence. Shared environmental risk factors include family related factors (e.g., neglect, parental stressors) and contextual factors in the surrounding community.

A recent study from Kahn, Deatre-Deckard, King-Casas and Kim-Spoon [71] has investigated the influences of both parenting and household environment on the intergenerational similarity in CU traits. They found a mediating role of hostile parenting in the association between parent and adolescent CU traits, especially in the context of high household chaos. They concluded that there is a heightened vulnerability to intergenerational transmission of these traits in a contest of household chaos that may exacerbate the effects of hostile parenting on CU traits during adolescence.

A recent meta-analysis showed that low family socio-economic status is associated with higher levels of children’s antisocial behaviours, and indicated that this relationship is stronger when CU traits are considered as outcome variables [72]. Previous researches [39, 73] consistently found that low family’s socio-economic status is the most important predictor of high CU traits. In the study by Muratori et al. [24] involving children with disruptive behaviour disorder, a higher level of socio-economic status at baseline was related to lower level of CU traits. Markowitz et al. [74] extended their research from the family to the neighborhood socio-economic context, by comparing the associations between high-CU traits and delinquency across adolescents living in high-, medium- and low-income neighborhoods in a non clinical sample. They confirmed the association between CU traits and delinquency; furthermore, they found that for high-CU individuals, living in high-income neighborhood hasn’t a protective effect on the likelihood to engage in delinquency compared to the moderate income, high-CU peer group. They also found that neighborhood context has an effect on the type, rather than the extent, of delinquency in which high-CU individuals are involved. In their sample CU traits were more strongly predictive of violent delinquency in low-income neighborhoods and of instrumental delinquency in high-income neighborhoods. Waller et al. [64], found that higher levels of exposure to violence predicted both moderate and high CU traits trajectories, while no associations were found between neighbourhood disorder and CU traits trajectory.

Previous studies have also investigated the relations between children/adolescents with high CU and peers group (for an extensive review see [34], paragraph “Parenting and Peer Risk Factors”). In Barker and Salekin’s study [75], experience of peer victimization at age 10 predicted CU traits at age 13 in children with a high score on a measure of irritability. Another characteristic of children and adolescents with CD and high CU traits may be a higher inclination to join with antisocial and delinquent peers, and consequently, to commit crimes in groups [76], compared to children and adolescents with CD but without CU traits [77]. However, Kerr et al. [78], suggest that adolescents with high CU traits could be less likely influenced by deviant peers group in putting into effect antisocial behaviours, but conversely, they could have a role in influencing antisocial behaviours of their group.

## Early signs and developmental trajectories

Although most of the studies about CU traits associated to CD are focused on children and adolescents, more recently the interest has been expanded to early childhood and toddler age, in order to detect early sings of CU behaviours. The possibility of detecting the origins of CU traits early in childhood is given by the emerging knowledge about individual differences in empathy and conscience as early as at the age of 2 or 3 years [79]. Consequently, several measures were developed or adapted to specifically assess possible precursors of CU traits (for a review see [80]). Aim of these studies is a timely identification of children at high risk of developing CD or antisocial behaviours, in a developmental phase with more malleable behaviour, when early interventions can be more effective [81]. In this earlier age, “CU behaviours” is a more adequate definition rather than “CU traits”, given the lesser evidences about their stability over time [67].

Higher CU behaviours in children as early as 3 year-old are related to lower guilt and empathy, more proactive aggression [67], and, perspectively, to CU traits in late childhood [67, 82, 83].

In his seminal work about the neurobiological basis of psychopathy, Blair [84] proposed that CU traits and behaviours emerge from a fearless temperament expressed through low arousal to others’ distress and punishment, leading to reduced learning about the consequences of harmful behaviours, and increasing the risk of CU behaviours. The measure of CU behaviours in children focuses on the presence of lack of empathy and guilt, in addition to a reduced emotional responsivity to others’ feelings or cues [21]. As a possible early indicator of CU behaviours in the adoption study mentioned above, Waller et al. [44] showed that the experimental observation of higher fearlessness and low affiliative behaviours at 18 months were related to higher CU behaviours referred by adoptive mothers at 27 months.

Recently, Goffin et al. [85] published the results of a long-term longitudinal study exploring early antecedents of CU traits and their evolution in typically developing children from toddler age to 12. They use observational measures to score guilt (in breaking a valued object situation), empathy (hurting the parent during play) and fearlessness in toddlers and preschoolers, and related these findings tolater parent-rated measures of CU traits and externalizing behaviours during scholar and pre-adolescent age. They found that children who were unconcerned to transgression (either breaking objects or hurting others) early in development exhibited higher CU traits in middle childhood. However, those links were moderated by child’s early temperament, being significant only in children high on fearlessness and not in fearful ones.

The developmental stability of CU traits has been investigated in several studies. In their review, Frick et al. [34] report a relative stability of CU traits from the age of 3–4 years-old according to parent rating. Although the CU trait levels tend to decreases across childhood and adolescence in a large number of children, those showing an elevated level of CU traits are at higher risk for keeping them in older ages. Other longitudinal studies have suggested that CU traits are relatively stable across childhood, and from childhood to adolescence. Dadds and colleagues [22] found moderate 1-year stability estimates for features of callousness (*r* = .55) in a community sample of young Australian children. Frick and colleagues [86] reported high 4-year stability estimates for parent ratings of callousness (interclass correlation = .71) from late childhood to middle adolescence. A more recent study of longitudinal invariance found that the rank-order stability of CU was moderate (*r* = .50, *p* < .001) during an 8-year period from childhood into adolescence among boys [87]. Fontaine, McCrory, Boivin, Moffitt, and Viding [73], using a person-centered approach (growth mixture modeling), showed that in children substantial decreases in CU traits across development were more common than substantial increases (see also Frick et al., [86]).

Also, Klingzell et al. [88] investigated joint trajectories of CU traits and conduct problems during early childhood in a community sample and found a close relationship between them (e.g. CU and conduct problems tend to decrease together or remain stable together) and with fearlessness and psychopathic traits. Despite the differences in stability across studies, these estimates of stability are comparable to those reported for others psychopathological constructs [89].

## Treatment

Conduct problems are usually highly impairing social, familial, and school functioning, and a management of this impairment is needed. Some specifiers, such as CU traits (or LPE DSM-5 specifier), as well as early age of onset of symptoms and comorbidities, should promote more timely referrals, assessments, and possibly an intensive treatment, and follow-ups.

The treatment of CP among children and adolescents with CU traits has been explored in depth, but so far, neither a proven psychological nor a definitive psychopharmacological treatment is available [90]. A recent systematic review by Hawes, Price, and Dadds [91] showed that CU traits were associated with increased risk for poor post-treatment outcomes. However, recent research has also suggested that children and adolescents with elevated CU traits are not “untreatable” and that they can improve with intensive treatments, tailored to the unique emotional, cognitive, and motivational styles [92].

Some recent and promising treatments specifically focused in ameliorating CU traits in the context of behavioural problems will be presented below and summarized in Table 1.

**Table 1:** Currently available psychotherapeutic treatments for children with conduct disorder and high CU traits

Name	Age range	Treatment target	Time range	Directed to (children, parents, teachers etc.)	Main references
CARES Module	3.5–8	Improvement of emotion recognition and labelling; enhancement of pro-social and empathic behaviour; increase of child’s frustration tolerance.	6 weeks	Children	Datyner et al., 2016
ERT	6–16	Enhancement of emotion recognition and interpretation; improvements of empathic abilities.	4 sessions (90 min each)	Children	Dadds et al., [94]
Mental Models	Adolescents	Increase positive emotion and reduce negative affect; improvement of decision-making skills; reduction of psychopathic features.	12 weeks	Children	Salekin et al., [96]
CP Program	7–14	Improvement of emotion recognition, especially anger; increase of child’s ability to cope with anger arousal; enhancement of perspective taking ability and problem solving skills; improvement of parenting skills.	12 months	Children and parents	Lochman and Wells, [97]; Muratori et al., [98]

Legend: *CARES* coaching and rewarding emotional skills, *ERT* emotion recognition training, *CP* coping power

Coaching and Rewarding Emotional Skills (CARES) Module is a brief emotional training program for assisting empathy and emotional development in young children with CP and CU traits. The CARES module was designed to be delivered to children between the ages of 3.5 and 8 years with non-normative levels of CU traits immediately after completion of parent management training, and when CP have been reduced to below clinical significance. CARES is the first targeted treatment designed for young children to ameliorate empathy-related deficits in processing negative emotions central to CU traits. The key treatment objectives of CARES are: (a) to enhance attention to critical facial cues signalling distress in child, parents and others, to improve emotion recognition and labelling; (b) improve emotional understanding by linking emotion to context, and by identifying contexts and situations that elicit child anger and frustration; (c) teach prosocial and empathic behaviour through social stories, parent modelling, and role play; (d) increase emotional labelling and prosocial behaviour through positive reinforcement; (e) and increase child’s frustration tolerance through modelling, role-playing, and reinforcing child’s use of learned cognitive-behavioural strategies to decrease the incidence of aggressive behaviours. Datyner et al. [93], in a case study, provided initial support for the short-term effectiveness of a brief adjunctive module to a PT intervention, to address empathy and emotions recognition deficits, and CU traits in children with CP + CU.

Emotion recognition training (ERT) [94] associated an emotion recognition training (ERT) and a PT intervention (Family Intervention for Child Conduct Problems). The authors showed that this association can lead to significant improvement in empathy and behavioural problems. Dadds et al. [94] pointed out that ERT could be added to usual intervention for clinically referred children with high CU traits. The ERT was partly based on the MindReading [95] program originally developed to train children with autism to accurately identify and interpret emotional expressions in interpersonal contexts. Mind Reading is a reference framework covering the entire spectrum of human emotions. Using the software, people can explore over 400 emotions, seeing and hearing each one performed by six different people. It includes an Emotion Library, a Learning Centre and a Game Zone. Studies showed that ERT produced significant improvements in affective empathy and CP in children with CD and high CU traits [94]. The association of ERT and PT seems to increase the level of reciprocated eye contact [91], and this should lead to enhancement of parent-child relationship.

Salekin et al. [96] tested a positive psychological intervention, named Mental Models, to reduce the behaviour problems in youth with higher CU traits. Using a combination of motivational techniques, cognitive behaviour training, and instruction on positive emotion, youth received 12 didactic sessions. The intervention was group-focused and included group sizes of four to 24 (mode = 6). The first group included all youth (*n* = 24), and the group size was then reduced to six individuals per group. The intervention involved a motivational component, whereby youth were motivated to participate in treatment. This component included some discussions on brain development and the new neural connections that can result through the process of active learning. Each week, many of the exercises were geared toward increasing positive emotions and positive ways of interacting with other individuals. Some exercises included writing assignments, entailing youth communicating their thoughts on strengths, ways to problem-solve, identification of emotions, and so forth. Youth were provided ways of thinking about these goals (models) and were also asked to generate their own ideas for setting goals and making plans for the future, to verbalize goals internally, and to mentally visualize the steps to accomplish a given goal, or to a better decision-making (including improving their choices, behaviour and citizenship within the institution). According to Salekin et al. [96], the intervention was effective at reducing interpersonal CU traits in youth, as well as improving their amenability to treatment.

Although these new interventions specifically developed for children with CD and elevated CU traits are promising, other more traditional treatments addressed for behavioural problems are available [25]. The Coping Power Program is a multi-component treatment program, delivered in a group setting, and has been developed using a contextual social–cognitive model as a conceptual framework for identifying intervention objectives [97]. The contextual social–cognitive model focuses on the contextual parenting processes, and on children’s sequential cognitive processing in the development and escalation of children’s behavioural problems. The CopingPower-child component includes 36 group sessions delivered over 12 months. In addition, 16 parent sessions are delivered over the same period. Parents also participate in small group sessions of five families; usually one parent per family attends the sessions. The child and his/her parent receive the treatment on the same day.

A recent study [98] found that the Coping Power program can lead to a significant decrease in CU traits, and this reduction was maintained at the 1-year follow-up evaluation. Authors hypothesized that the specific intervention on parenting practices promoted the decrease in CU traits in children; and that Coping Power group therapy can improve emotion sharing with peers, children’s understanding of other people’s emotions, and consequently has a beneficial influence on their empathy as well [99].

Regarding medications, a recent review [100] suggests that methylphenidate and risperidone present the largest effects on aggression, while other antipsychotics show clinical efficacy on CD, but this evidence is mainly supported by open label trials. There is some low quality evidence to support a small effect of mood stabilizers and other agents. Nevertheless, antipsychotic drugs are associated with meaningful adverse events (namely metabolic disturbances such as obesity, diabetes, dyslipidemia [101, 102] that requires great caution in their use in childhood. No drug treatments specifically work for CU traits, and they may be predictors of poor response to pharmacological treatments in CD, although data are still inconclusive [100, 103]. We have recently shown that a pharmacotherapy may constitute an important added value to psychosocial interventions in DBD, improving emotional dysregulation but not CU traits [104].

## Discussion

The aim of this paper was to provide a brief review of the main features of childhood psychopathy, and specifically on the CU traits, which represent its affective dimension (e.g. which encompasses multiple deficits in emotional processing). Paediatricians manage the health of children and adolescents since their birth, including behaviour and mental health issues, and, usually, they are the primary care specialists who provide the first contact for children and their families. Therefore, they could represent the first step in identifying the presence of CU traits, and the possible developmental pathways to antisocial behaviours, and in promoting early interventions. Additionally, considering the caveats in clinical assessment, paediatricians may help to identify the “persistently over at least 12 months and in more than one relationship or setting” DMS-5 criterion.

This and other reviews [21, 26, 46, 72, 79] clearly demonstrate that CU traits are highly heritable, that a timely identification is possible, since early years, and that they are associated with identifiable environmental risk factors. Also, they can be targeted with specific interventions, and, to some extent, partially modifiable. Well designed longitudinal studies, both naturalistic and intervention, mainly in clinical samples, are needed to further delineate developmental trajectories, their predictors, and how CU traits alone or interacting with behavioural symptoms predict the outcomes.

Implications are straightforward. A thorough knowledge of family interactions, easily available for paediatricians, is crucial for early identification as well for a therapeutic alliance with parents and children. Some of the existing instruments can be possibly implemented in paediatric settings [26], even if an easier and quicker questionnaire, more suitable for a population based screen, is still lacking. Further researches should fill this gap.

The identification of behavioural problems and CU traits during childhood may be helpful to promote early interventions. As pointed out in the paper, there are currently available some promising treatment programs, specifically addressed for children and adolescents with CD and high level of CU traits (e.g., Coping Power Program, CARES, ERT, Mental Models), whose main targets are the improvement of emotion recognition, the enhancement of prosocial and empathic behaviour, and the improvement of parenting skills. Well designed, randomized trials should be the next step for evidence based treatments directly targeting CU traits.

## Conclusion

As a summary, we conclude that a strong genetic liability, in interaction with parenting and relevant environmental factors, can lead to elevated levels of CU traits; they can be detected in early childhood, may remain stable along the adolescence, but a possible decrease following intensive and specialized treatment is possible. We maintain that a strict collaboration among paediatricians, child psychiatrists, clinical psychologists and all other professionals devoted to child mental and physical health is needed to early detection and management of these severely impaired patients.

## Abbreviations

APSD: Antisocial process screening device
CARES: Coaching and rewarding emotional skills
CD: Conduct disorder
CP: Conduct problems
CPTI: Child problematic traits inventory
CU: Callous unemotional
DSM-5: Diagnostic and statistical manual of mental disorder- 5 edition
ERT: Emotion recognition training
ICU: Inventory of callous unemotional traits
LPE: Limited pro-social emotion
PCS: Childhood Psychopathy Scale
PT: Parent training
UNSW system: University of New South Wales system
YPI: Youth psychopathic traits inventory
